# A protocol for adapting a clinical practice guideline for the treatment of paediatric asthma for the Egyptian Pediatric Clinical Practice Guidelines Committee

**DOI:** 10.1002/gin2.12011

**Published:** 2024-03-13

**Authors:** Ashraf Abdel Baky, Ahmed Youssef, Lamis Elsholkamy, Mona Saber, Nahla Gamal, Nanies Soliman, Yasser Amer

**Affiliations:** ^1^ Pediatrics Department, Faculty of Medicine Ain Shams University Cairo Egypt; ^2^ Pediatrics Department MTI University Cairo Egypt; ^3^ Egyptian Pediatric Clinical Practice Guidelines Committee Cairo Egypt; ^4^ General Organization for Teaching Hospitals and Institutes Cairo Egypt; ^5^ Pediatrics Department, King Khalid University Hospital King Saud University Medical City Riyadh Saudi Arabia; ^6^ Clinical Practice Guidelines and Quality Research Unit, Quality Management Department King Saud University Medical City Riyadh Saudi Arabia; ^7^ Research Chair for Evidence‐Based Health Care and Knowledge Translation King Saud University Riyadh Saudi Arabia; ^8^ Department of Internal Medicine, Ribeirão Preto Medical School University of São Paulo (FMRP‐USP) Ribeirão Preto São Paulo Brazil; ^9^ Adaptation Working Group, Guidelines International Network Perth UK

**Keywords:** adapted ADAPTE, asthma, child healthcare, guideline adaptation, guideline protocol, paediatrics, practice guidelines

## Abstract

**Introduction:**

Paediatric asthma is a prevalent and chronic respiratory condition affecting children worldwide. This protocol outlines the methodology for developing a clinical practice guideline (CPG) for the management of paediatric asthma. The goal of this guideline is to provide evidence‐based recommendations for healthcare professionals, improving the quality of care and health outcomes for paediatric patients with asthma.

**Methods:**

We will use the ‘Adapted ADAPTE’ adaption method, a systematic approach to adapt existing guidelines, which consists of three phases (setup, adaptation and finalisation phases), nine modules and 24 steps, with adaptations to the process and tools to suit the Egypt healthcare context and resources.

**Questions:**

Clinical questions were prepared using the patient population, interventions, professionals, outcomes and healthcare context (PIPOH) model. This CPG protocol addresses critical clinical questions at the heart of effective asthma management in children. It focuses on the diagnosis of paediatric asthma, considering age‐specific clinical presentations and diagnostic tests, assessment of asthma severity, control medications like inhaled corticosteroids, long‐acting β‐agonists and others, with an emphasis on appropriate tools and criteria. The CPG also delves into the realm of long‐term control treatment, exploring the effectiveness and safety of pharmacological and nonpharmacological interventions, while considering individualised needs. Furthermore, it examines strategies for monitoring and adjusting treatment plans over time, ensuring optimal care. These clinical questions form the foundation of the CPG, facilitating evidence‐based care delivery and enhancing health outcomes for paediatric asthma patients.

## Highlights

- Asthma is a high‐priority health topic for paediatrics and child healthcare in Egypt.
- This national guideline adaptation project is part of the Egyptian Pediatric Clinical Practice Guidelines Committee (EPG).
- The EPG Guideline Adaptation Group will use the ‘Adapted ADAPTE’ Methodology.
- The authors will consider both the ADAPTE‐based and GRADE‐based approaches.
- The AGREE II Instrument will guide the selection of high‐quality appraised source guidelines.
- The RIGHT‐Ad@pt Tool will guide the reporting of the adapted guideline.

## 1 INTRODUCTION AND SCOPE

Bronchial asthma is a chronic inflammatory airway disease that occurs worldwide with significant ethnic and regional variability resulting in long‐term morbidity and mortality.^1^ Recurrent attacks, multiple exacerbations and poor clinical control have a huge impact on the physical and mental health of children.^2^

Bronchial asthma is one of the most common noncommunicable diseases throughout the life span, impacting more than 350 million children, adolescents and adults worldwide.^3^

It mainly occurs in young children and has an 8%–28% incidence and prevalence globally, and it is increasing yearly.^4^ In Egypt, the prevalence of asthma among school children in the Nile Delta region is about 7.7%^5^ with probably underdiagnosis and undertreatment, particularly among children from less wealthy families.^6^ Other studies reported an incidence of 9.4% in 11–15‐year‐old adolescents.^7^ All these figures highlight the importance of setting a standardised approach in the management of children with chronic asthma with good interaction between healthcare providers and patients.

Until recently, asthma diagnosis had been made very simplistically predominantly from a clinical history and examination, and often a trial of medication such as short‐acting bronchodilators. The limitations of this approach have become increasingly apparent with evidence of inappropriate overdiagnosis, underdiagnosis and misdiagnosis. Although there is no gold standard single test to make a diagnosis of asthma, there are several objective tests that can be used to support the diagnosis including physiological measures such as obstructive spirometry associated with bronchodilator reversibility and airway hyperresponsiveness.^8^

The current clinical treatment goal for childhood asthma is to achieve complete control of asthma and to carry out its long‐term maintenance, including controlling the clinical symptoms and improving the inflammation and pathophysiological indicators.^9^

A stepwise approach in asthma management depends on the severity and frequency of attacks. Pharmacotherapy generally consists of inhaled corticosteroids, long‐acting β‐2 agonists, mast cell stabilisers as well as other drugs like sustained‐release theophylline and biological therapy which is reserved for severe persistent cases. Allergic rhinitis, eosinophilic chronic rhinosinusitis and chronic obstructive pulmonary disease are also important conditions to be considered in asthma therapy.^9^

There is a high variability in the quality of clinical practice guidelines (CPG) in general and of the paediatric asthma guidelines in particular globally and locally.^10^, ^11^, ^12^

In 2019, the Scottish Intercollegiate Guidelines Network (SIGN) and the British Thoracic Society (BTS) updated the British guideline on the management of asthma (SIGN 158) including the recommendations for the best practice in the management of asthma in adults, including pregnant women and children. This CPG presented recommendations for asthma diagnosis, monitoring, supported self‐management, nonpharmacological management, pharmacological management, inhaler devices, management of acute asthma, difficult asthma, asthma in adolescents, asthma in pregnancy, occupational asthma, organisation and delivery of care and provision of information. It included a set of CPG implementation tools as well as a quick references guide, clinical algorithm, summary of management, decision aids and patient information.^13^

Currently, there is an ongoing project for updating this SIGN 158 CPG jointly by SIGN, BTS and the National Institute for Health and Care Excellence (NICE) to produce a UK‐wide guideline on asthma diagnosis and monitoring and chronic asthma management that is expected to be published on 30 October 2024. We will follow up on the official page of the asthma CPG on both websites SIGN and NICE for the publication of the updated joint CPG.

The Egyptian Pediatric Clinical Practice Guidelines Committee (EPG) was founded in 2018 and was launched as the first paediatric national CPG adaptation programme using an evidence‐based CPG adaptation methodology in Egypt.^14^, ^15^ The EPG has produced more than 30 adapted CPGs in six consecutive waves with the contribution of different EPG subspecialty groups (Link: http://epg.edu.eg/).

Adaptation of CPGs involves modifying existing CPGs to suit specific regional, cultural or systemic needs in healthcare. This process ensures that CPGs are more relevant and applicable to the target population, enhancing their effectiveness in local clinical settings. The benefits include improved patient outcomes due to more tailored treatments, and efficient resource utilisation, as the adapted CPG aligns with local healthcare infrastructure and practices. This approach bridges the gap between universal medical standards and practical, localised healthcare delivery.^16^, ^17^

A recent review showed that six of the formal CPG adaptation methodological frameworks were used in the Eastern Mediterranean Region including ADAPTE, CAN‐IMPLEMENT, Adapted ADAPTE, RAPADAPTE, GRADE‐ADOLOPMENT and KSU‐Modified‐ADAPTE.^14^

Moreover, the World Health Organization Europe Regional Office (WHO/Europe) has issued a new handbook in 2023 to assist countries in adopting, adapting and contextualising evidence‐based CPGs to their own national settings and systems^18^ and the WHO Eastern Mediterranean Regional Office (WHO/EMRO) has launched the Evidence and data to policy platform and a series of face‐to‐face and virtual technical workshops on developing, adapting, implementing and reviewing CPGs to support the National Guidelines Programme in Egypt in collaboration with the WHO Country Office of Egypt.^19^

Additionally, WHO/EMRO has highlighted the importance of the increasing use of living evidence and living guidelines.^20^

### 1.1 Scope of the CPG

The scope of this CPG, including the key questions, was identified and detailed using the PIPOH model including Patient population including the disease characteristics (P), Intervention(s) of interest (I), Professionals and clinical specialties of the target audience of this CPG (P), Outcomes to be considered or the purpose of this CPG (O) and Healthcare setting and context (H). The PIPOH model was first proposed by the original ADAPTE resource toolkit.^21^

The group decided to use the PIPOH, which is one of the tools included in the ADAPTE‐based CPG adaptation methodological frameworks (e.g. original ADAPTE, Adapted ADAPTE and KSU‐Modified‐ADAPTE) rather than the PICO model, that includes the same components except the Comparison (C) was not applicable to or included in this model.

## 2 GUIDELINE QUESTIONS AND OUTCOMES

**Figure 1:**
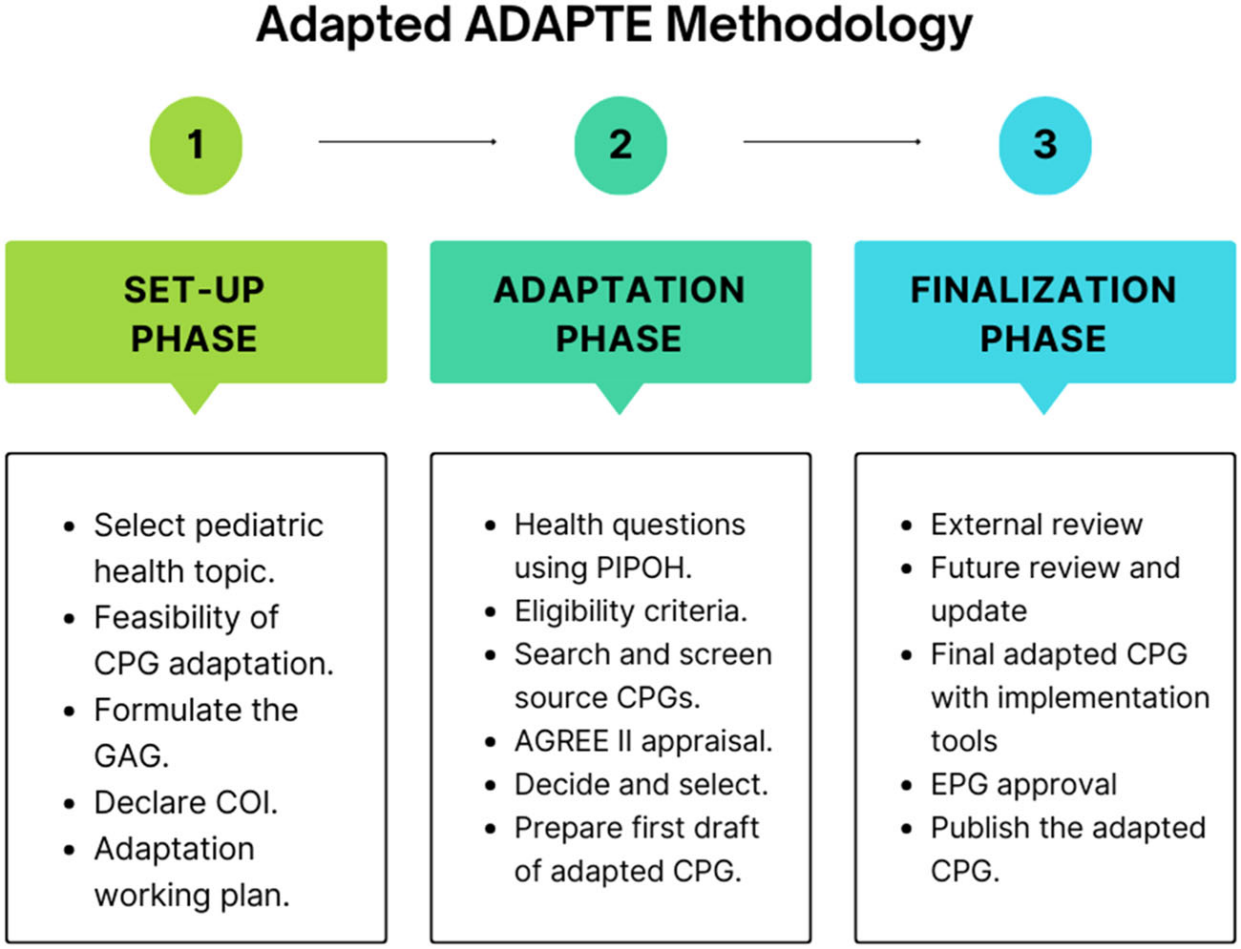
Summary of the Adapted ADAPTE methodology. AGREE II, Appraisal of Guidelines for Research and Evaluation Instrument Version II; COI, conflicts of interest; CPG, clinical practice guideline; EPG, Egyptian Pediatric Clinical Practice Guidelines Committee; GAG, guideline adaptation group; PIPOH, patient population, interventions, professionals, outcomes and healthcare context.

The Guideline Adaptation Group (GAG) identified and prioritised 14 health questions as presented in Table 1. The prioritisation processes for questions included a formal voting process based on the common variability of practice in asthma management in Egypt, various healthcare needs and patient values and preferences. These included questions on the diagnostic criteria, severity (grading), treatment (control), monitoring and comorbidities in children with asthma using the PIPOH tool. The final set of questions was compared to the questions proposed by the latest accessible BTS/SIGN and GINA CPGs as part of the preliminary literature review. As the GAG prioritised this set of health questions based on the Egyptian healthcare context, the adaptability of these questions in light of evidence to a different healthcare context is not applicable which could be a potential limitation (Figure 1).

**Table 1:** Health questions.

Patient population and interventions	Prioritised outcomes
*Severity (grading)*	
1. What is the best practice in the diagnostic criteria of children aged 5 years or less with asthma including history of wheezing or cough without apparent respiratory infection, other allergic diseases, clinical history of symptoms in response to exercise and assessing clinical improvement after using low‐dose inhaled corticosteroids (ICS) within 2–3 months?	Diagnosis of asthma in children 5 years old or less.
2. What is the best practice in the diagnostic criteria of children aged 6–12 years with asthma, including a history of wheezing or cough without apparent respiratory infection, other allergic diseases, clinical history of symptoms in response to exercise, spirometry at diagnosis and assessing the clinical improving after using low‐dose ICS within 2–3 months?	Diagnosis of asthma in children 6–12 years old.
*Treatment (control)*	
3. What is the best practice for assessing asthma severity in children including analysing daytime symptoms and their effect on activity, nighttime symptoms and pulmonary function tests)?	Grading of asthma symptoms.
4. What is the best practice for the initial management based on asthma severity including low‐dose ICS, long‐acting β‐2 agonists (LABAs) and β2 agonists?	Asthma symptom control, severity of asthma attacks.
5. How often should a child be reviewed for asthma control after initial treatment and after exacerbation including the analysis of symptom control over the last 4 weeks, spirometry at diagnosis, at starting of treatment and after 3 months, periodically and the frequency of reliever use?	Assessing the treatment control.
6. How to assess asthma control in children 5 years old or less including analysing daytime symptoms and their effect on activity, nighttime symptoms and SABA reliever medication needs?	Assessing the treatment control.
7. How to assess asthma control in children 6–12 years including the analysis of symptom control over the last 4 weeks, spirometry at diagnosis, at starting of treatment, after 3 months, periodically and the frequency of reliever use?	Assessing the treatment control.
8. Which marker(s) is(are) the most effective for monitoring current asthma control in children with asthma of less than 5 years and 6–12 years of age including symptom scores, lung function test, sputum and blood (e.g. eosinophil count)?	Assessing the treatment control by asthma symptom control, number of asthma attacks, severity of asthma attacks, adverse effects, treatment adherence and lung function test.
9. Which interventions (avoidance or reduction of exposure to environmental factors) in the home, school and outdoor environment improve asthma control and prevent or reduce the severity of asthma attacks?	Assessing the treatment control.
10. What is the step‐up approach in the treatment of not adequately controlled asthma in different age groups of children?	Assessing the treatment control of the symptoms in children 5 years old or less and aged 6–12 years.
11. When to step up the treatment in children? After confirming the diagnosis, monitoring inhaler technique, adherence, allergen exposures and/or comorbidities?	Assessing the treatment control of the symptoms in children 5 years old or less and aged 6–12 years. Deciding when to step up or step down in treatment.
12. When to step down the treatment? After monitoring the asthma symptoms control for how long?	Assessing the treatment control of the symptoms in children 5 years old or less and aged 6–12 years. Deciding when to step up or step down in treatment.
*Monitoring*	
13. In people with asthma (<6, 6–12), which individual, or combination of, characteristic(s) effectively predict(s) future loss of control and/or future risk of attacks? These may include symptom pattern, asthma control, asthma severity, previous history of attacks, atopy (including sensitisation, comorbid allergic conditions, family history), treatment adherence and behaviours and social deprivation.	Number of asthma attacks, frequency of asthma attacks.
*Comorbidities*	
14. What is the impact of the management of asthma comorbidities (like allergic rhinitis, obesity and exercise‐induced asthma) on asthma severity?	Assessment of asthma severity (grading) in children with one or more of these comorbidities.
	Better control of asthma symptoms.
	Additional measures to be considered are as follows:
	*For allergic rhinitis*: Assess itching, sneezing, nasal obstruction, ability to breathe through the nose and medication to control nasal symptoms (e.g. intranasal corticosteroids). *For obesity*: Check age BMI, dietary history and physical activity. *For exercise*‐induced bronchospasm: Regular training, warm up and weight reduction; inhaled corticosteroids and β‐2 agonists before exercise.

Abbreviations: BMI, body mass index; ICS, inhaled corticosteroids; LABAs, long‐acting β‐2 agonists.

### 2.1 Patient population

Children before the age of 12 years, diagnosed with asthma by a physician with or without comorbidities (e.g. allergic rhinitis, obesity and exercise‐induced asthma). Two age subgroups were considered: children aged 5 years or less and children aged 6–12 years.

Children with acute asthma exacerbations will be excluded from the patient population of this CPG as this patient category was included in an earlier EPG CPG adaptation project that was already finalised and released in the first wave of EPG national CPGs initiative.^14^

### 2.2 Interventions

Diagnostic criteria, control, monitoring and managing comorbidities considering different age groups and different grades of severity of paediatric asthma.

### 2.3 Professionals and clinical specialities

General practitioners, general paediatricians, family medicine physicians, paediatric pulmonologists, paediatric allergy, immunology physicians, clinical pharmacists, nurses and clinical nutritionists.

### 2.4 Outcomes

The outcomes were prioritised based on their impact on the health, values and preferences of children with asthma (e.g. assessment of asthma control and need for hospitalisations).

#### 2.4.1 Primary outcomes

- Improvement of morbidity and mortality.
- Improve the control of asthmatic attacks.

#### 2.4.2 Secondary outcomes

- Improve normal growth pattern and nutritional status.
- Decrease hospitalisations.
- Decrease school absence.
- Promote psychological well‐being.

### 2.5 Healthcare setting

Primary, secondary and tertiary care services include governmental, nongovernmental sectors, hospitals and outpatient clinics in Egypt.

## 3 METHODS

The process of CPG adaptation will follow the Adapted ADAPTE, a formal methodology for CPG adaptation, which was initially developed based on the original ADAPTE resource toolkit to adapt high‐quality CPGs to suit the local context and health system in Alexandria University Hospitals especially the Alexandria University Children's Hospital and later was used at a national level by the EPG national pediatric guidelines initiative.^14^, ^15^, ^21^

The ‘Adapted ADAPTE’ is designed to enhance the usability of the original ADAPTE resource toolkit for guideline adaptation. The adapted methodology focuses on clarity, simplicity and practicality, aiming to minimise duplication and resource expenditure. The process involves a preliminary study to understand current practices and then applies the ‘Adapted ADAPTE’ methodology, which includes three phases, ‘set‐up’, ‘adaptation’ and ‘finalisation’, three modified tools and three new tools derived from the original ADAPTE framework, alongside alternatives for four ADAPTE steps to improve utilisation. This methodology was designed to customise and streamline the process of adapting CPG to specific local healthcare contexts, particularly in resource‐limited regions.^21^

The classification of the quality of evidence and strength of recommendations will be guided by the Grading of Recommendations Assessment, Development and Evaluation (GRADE) system and the final adapted CPG will be reported using the Reporting Items for practice Guidelines in HealThcare statement extension for CPG adaptation (or the RIGHT‐Ad@pt Tool).^22^, ^23^

The RIGHT‐Ad@pt Tool streamlines adapting existing clinical guidelines to local contexts, ensuring they remain relevant, evidence‐based and resource‐efficient while involving stakeholders and maintaining transparency in reporting. It is divided into 7 sections, 27 topics and 34 items. The seven sections include (i) basic information; (ii) scope; (iii) rigour of development; (iv) recommendations; (v) external review and quality assurance; (vi) funding, declaration and management of interest and (vii) other information.

In writing this protocol, the authors were guided by the guidelines article types, descriptions and formats provided by the Journal of Clinical and Public Health Guidelines in addition to the published work of Xun et al on protocols for CPGs.^24^ There are currently no suggested reporting checklists for guideline protocols by the EQUATOR Network.

### 3.1 Prospective registration

This EPG CPG adaptation project was registered in the ‘Practice guideline REgistration transparency (PREPARE)’ platform under the registration number (PREPARE‐2023CN114). PREPARE is hosted by the World Health Organisation Collaborating Centre for Guideline Implementation and Knowledge Translation, Evidence‐Based Medicine Center, Lanzhou University, China.^25^, ^26^

### 3.2 The Guideline Adaptation Group (GAG)

The GAG will be formed from paediatricians with expertise in managing patients with asthma and other clinical stakeholders as relevant will be recruited including paediatric allergy and immunology, paediatric pulmonology and clinical pharmacists.

Additionally, a CPG methodology advisory subgroup will support and guide the CPG adaptation process. All GAG members will sign a conflicts of interest (COI) statement, the core group members will be reported with this protocol and any future members that will be further recruited as needed will be reported in the final adapted CPG full document and publication.

The clinical chair of this GAG is also the EPG chairman based on his academic and clinical expertise in addition to his experience with using the Adapted ADAPTE methodology in previous EPG CPG adaptation projects.

### 3.3 Literature searches

A comprehensive and systematic literature search for published eligible paediatric asthma CPGs based on the identified PIPOH, health questions and inclusion and exclusion criteria.

Three categories of databases, repositories and websites will be searched for potential asthma CPGs.

First, bibliographic databases include PubMed, Google Scholar and the Egyptian Knowledge Bank to identify CPGs published as full‐text articles in peer‐reviewed medical or healthcare journals. Published traditional Cochrane or non‐Cochrane systematic reviews that are preappraised using ‘A MeaSurement Tool to Assess systematic Reviews’ (AMSTAR) or AMSTAR2 and systematic reviews and meta‐research of paediatric asthma CPGs that are preappraised using the Appraisal of Guidelines for Research and Evaluation II (AGREE II) Instrument will be considered as well.^27^, ^28^, ^29^

Second, CPG databases include the Guidelines International Network (GIN) International library and registry, PREPARE platform, SIGN, NICE, ECRI and DynaMed. Additionally, open recommendations maps like BIGG‐REC (PAHO/WHO) and GRADE EtD's and Guidelines (GRADEpro/GDT) will be searched as well.

Third, related professional speciality and subspeciality societies' websites include the Global Initiative for Asthma (GINA), British Thoracic Society (BTS), American Thoracic Society (ATS), American College of Allergy, Asthma and Immunology (ACAAI), World Allergy Organization (WAO), European Academy of Allergy and Clinical Immunology (ECAAI) and American Academy of Allergy, Asthma and Immunology (AAAAI).

### 3.4 Evidence and guideline eligibility

#### 3.4.1 Inclusion and exclusion criteria for the source CPGs

Eligible paediatric asthma source CPGs will be those that are (i) de‐novo developed using the GRADE system with a clear and detailed report of the methodology of development and preferably of the GRADE Evidence‐to‐Decision (EtD) frameworks,^30^ (ii) including the CPG development group, (iii) National and international CPGs will be considered, (iv) published within the last 5 years and (v) language of publication is English or Arabic.

Exclusion criteria will include nonevidence‐based CPGs, single‐authored CPGs, CPGs published before 2019, CPGs published in languages other than English and Arabic and CPGs that are adapted from other source CPGs.

### 3.5 Guideline appraisal

#### 3.5.1 The AGREE II instrument

The AGREE II will be used to appraise the eligible source paediatric asthma CPGs methodologically and critically. Each source CPG will be appraised by four members of the GAG that have received prior capacity building and calibration on appraising CPGs using AGREE II using its online platform: My AGREE PLUS. Standardised domain scores of the six AGREE II domains, the two overall assessments, will be considered with a focus on Domain 3 (rigour of development) and a minimum cut‐off for domain scores or ratings of recommended CPGs of 60% to define a high‐quality CPGs that was suggested in previously published AGREE II appraisals.^31^ Appraised CPGs will then be classified into high quality (Domain 3 scores >60%), low quality (Domain 3 score <40%) and moderate quality (Domain 3 score 40%–60%) and a CPG content review and analysis will be conducted to guide the selection of the appraised CPGs.

The GAG may choose not to conduct an assessment of the certainty of evidence and evidence synthesis if it finds recommendations with evidence base that are applicable and acceptable in the context of implementation.

### 3.6 Recommendation grading

The strength of recommendation classifications will follow the GRADE system that includes two types of either strong or conditional (weak) recommendations that in turn will affect the articulation of each recommendation statement.^30^

### 3.7 Methods for formulating recommendations

The GAG will conduct an informal consensus process after reviewing the recommendations of the source high‐quality CPG(s) considering the nation‐wide applicability, acceptability and implementability in line with the national health system in Egypt after discussion of the relevant contextual factors from the GRADE EtD's of the source CPGs (e.g. benefits and harms of interventions, resource use, equity, acceptability and feasibility).

### 3.8 The external review group

The external review will include two types of review (clinical content review and methodology review). This group will comprise experts in general paediatrics, family medicine, paediatric pulmonology, paediatric allergy and immunology, clinical pharmacy, nursing care and clinical nutrition. Additionally, we will attempt to recruit a patient representative, patient advocate or family representative of a child with asthma to address patient values and preferences and ensure inclusiveness and equity.

### 3.9 COI management

3.10

All contributors to this CPG project will declare their COIs and handle any potential COIs guided by the nine core principles for disclosure of interests and management of conflicts in guidelines that were developed by GIN.^32^

### 3.11 Funding sources and funder role

There is no funding associated with this CPG adaptation project and all contributors are voluntary and academic researchers.

## AUTHOR CONTRIBUTIONS

*Conceptualization, methodology, and supervision*: Ashraf Abdel Baky and Yasser Amer. *Writing the first draft of the manuscript*: Yasser Amer. *Writing, review, and editing*: Ashraf Abdel Baky, Ahmed Youssef, Lamis Elsholkamy, Mona Saber, Nahla Gamal, Nanies Soliman, and Yasser Amer (members of the guideline adaptation group). *Visualization*: Yasser Amer. *Funding Acquisition*: Not applicable. All authors have reviewed and agreed to the submitted version of the manuscript.

## CONFLICT OF INTEREST STATEMENT

The authors declare no conflict of interest.

## ETHICS STATEMENT

Not applicable.

## Data Availability

The data that support the guideline protocol have been made available in the table, figure and supplementary file of this article. Further details could be made available from the authors upon reasonable request to the corresponding authors.
